# 4-Methoxypicolinic Acid *N*-Oxide: One of the Shortest Hydrogen Bonds Known Characterized by Neutron Diffraction, Inelastic Neutron Scattering, Infrared Spectroscopy, and Periodic DFT Calculations

**DOI:** 10.1021/acsomega.4c05344

**Published:** 2024-08-29

**Authors:** Jernej Stare, Jože Grdadolnik, Sax Mason, Alberto Albinati, Juergen Eckert

**Affiliations:** † Theory Department, National Institute of Chemistry, Hajdrihova 19, SI-1000 Ljubljana, Slovenia; ‡ Institut Laue-Langevin, 6 rue Jules Horowitz, BP 156, 38042 Grenoble Cedex 9, France; § CNR-ICCOM, Sesto Fiorentino and University of Milan, Via Madonna del Piano, 50119 Milan, Italy; ∥ Department of Chemistry and Biochemistry, Texas Tech University, P.O. Box 41061, Lubbock, Texas 79409-1061, United States

## Abstract

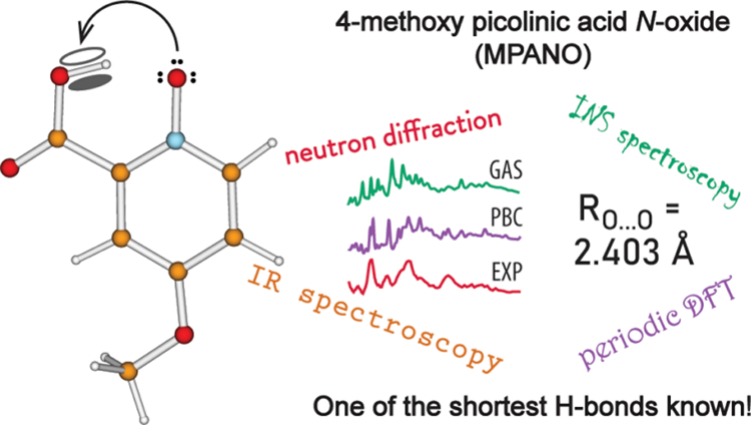

The present work focuses on the case of an extremely short intramolecular O–H···O hydrogen bond (H-bond) found in 4-methoxypicolinic acid *N*-oxide (MPANO). The donor···acceptor separation of 2.403 Å makes the H-bond in MPANO one of the shortest H-bonds known. We elucidated the structure and dynamics of the H-bond by two neutron-based techniques, namely, single-crystal diffraction and inelastic scattering (INS) vibrational spectroscopy. We also utilized conventional infrared (IR) spectroscopy as well as quantum chemical computations on isolated and periodic models. Both the protiated and deuterated variants of MPANO were investigated by INS and IR. All the methods used unequivocally confirm the existence of an extremely short, asymmetric H-bond, with the proton located near yet off the midpoint. The main relevant feature of the IR spectrum is an extremely broad, complex, and red-shifted OH (OD) stretching band spanning between 1800 and 500 cm^–1^ and centered at about 1360 cm^–1^, which indicates the presence of extensive anharmonicity and coupling with other H-bond modes. Of the modes characteristic of H-bond dynamics, only the out-of-plane OH (OD) bending can clearly be detected in the INS spectra; it has a relatively high frequency indicative of the strength of the H-bond. The computed structure is in excellent agreement with the diffraction measurement when periodicity is taken into account. The calculated harmonic frequencies show a reasonable match with the observed spectral features, whereby the assignment of the IR and INS spectra is facilitated. The hydrogen stretching frequency, however, appears to be significantly overestimated, on account of the limitations of the harmonic approximation and the complex nature of the short H-bond.

## 1 Introduction

Hydrogen bonding (H-bonding) is one of the most important interatomic (and/or intermolecular) interactions, as it plays a substantial role in the structure and functionality of a wide range of chemical systems, including biomolecules and materials. The unique and intriguing properties of H-bonding have long been the subject of extensive investigations using a wide array of experimental and computational research techniques. Despite the impressive amount of information that has been collected, many characteristics of the H-bond remain poorly understood. This is particularly true for the class of short (strong) H-bonds, for which most of the commonly observed effects are expressed in a radically different and often disproportional manner. While short H-bonds are interesting in their own right because of their intriguing features such as the enormously red-shifted and broadened proton stretching bands in the infrared spectra, research on very short H-bonding received a strong impetus when Cleland and Kreevoy suggested that this short H-bond, now called a “low-barrier H-bond”, may play a vital role in certain enzymatic reactions.^1^ Their proposal has been widely debated and investigated both theoretically and experimentally,^2−4^ and the related research extended well beyond biomolecular systems. Nonetheless, studies of the nature and role of short H-bonding have always been somewhat limited on account of a shortage of examples and also because of difficulties in the interpretation of experimental observables. This can often be a challenging task, for example, finding the precise location of the proton or assignment of infrared spectral bands.

Among the techniques capable of precisely probing the characteristics of short H-bonds, those based on the interaction with neutrons represent a viable but to date only rarely exploited approach. Neutron scattering techniques are not easily accessible because they are only available at special facilities and often impose considerable requirements on the size and quality of samples but can provide unique insights into the structure and dynamics of matter with H-bonds being a prime example. The use of neutron diffraction (ND) given the large coherent scattering cross-section of deuterium atoms makes it possible to determine the location and thermal displacement (ADPs) of D in the H-bond much more precisely than by X-ray diffraction (which is sensitive to the electron density and bonding effects). Vibrational spectroscopy by inelastic neutron scattering, on the other hand, can circumvent many of the issues in optical spectroscopy (e.g., selection rules) on account of the simplicity of the neutron interaction with the atomic nuclei.^5,6^

The present work also illustrates the power of neutron techniques when used for the characterization of the structure and vibrational dynamics of an extremely short H-bond in the crystalline solid state by a combination of neutron diffraction, inelastic neutron scattering vibrational spectroscopy (INS), and periodic quantum chemistry calculations capable of elucidating fine structural details. The latter is particularly important for INS spectroscopy since both frequencies and intensities can readily be obtained from density functional theory (DFT) calculations. This property can be critical in reaching an understanding of H-bond dynamics when making use of the isotopic substitution of H by D in the H-bond in the experimental INS spectra. The reason for this utility is that H and D possess dramatically differing neutron scattering properties on account of the difference in the neutron–nuclear interaction, which in turn results in pronounced changes in intensities of a vibrational band. This can be used to readily identify vibrational modes of the H-bond proton, and their coupling to other modes can be readily identified.

**Scheme 1:**
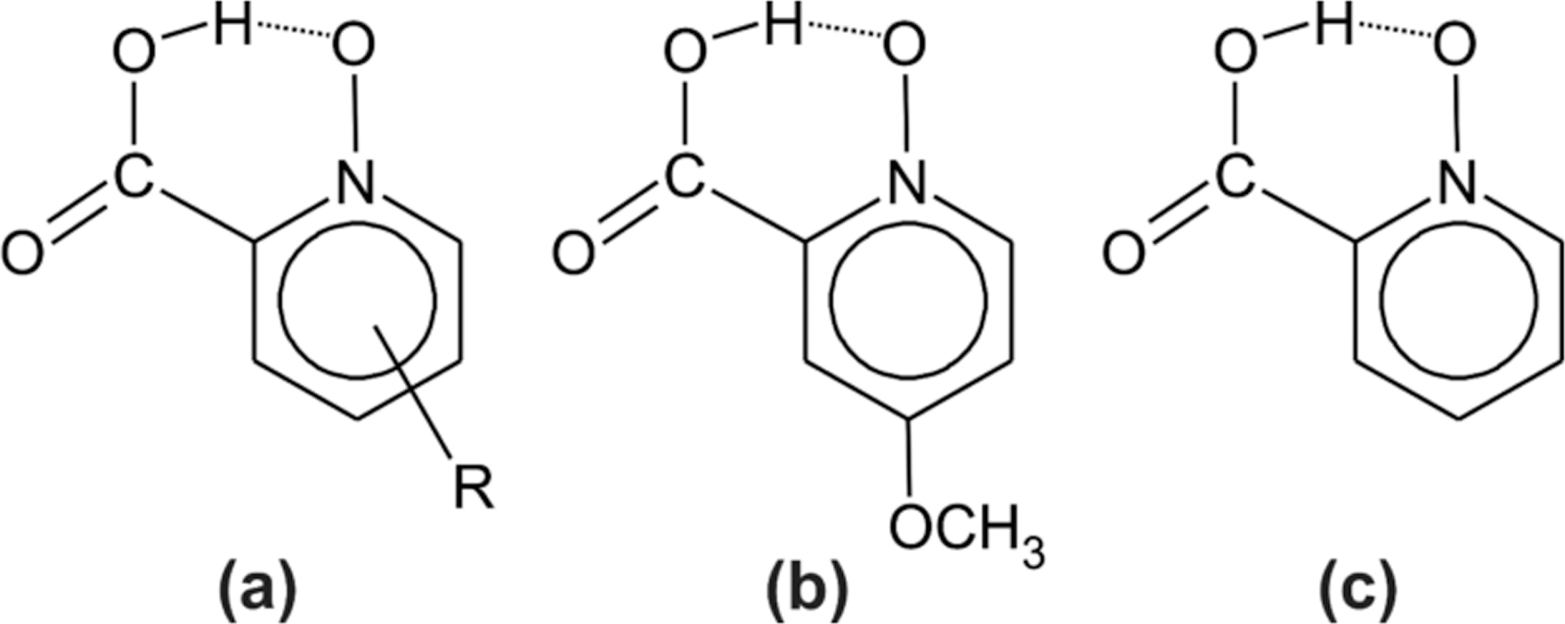
(a) General Scheme of the Substituted Picolinic Acid *N*-Oxide Group Members, (b) 4-Methoxypicolinic Acid *N*-Oxide (MPANO), Studied in This Work, and (c) Unsubstituted Picolinic Acid *N*-Oxide (PANO)

The subject of our investigation is 4-methoxypicolinic acid *N*-oxide (MPANO), a member of the group of picolinic acid *N*-oxide (PANO), and its substituted analogs (Scheme 1), all of which possess a very short intramolecular O–H···O H-bond (*R*_O···O_ < 2.5 Å).^7^ Unlike the best known examples of short H-bonds such as acetylacetone,^8,9^ hydrogen phthalate,^10,11^ hydrogen maleate,^12,13^ cobalt tris chelates of 2-aminoethanol,^14^ or the Zundel cation ([H_2_O···H···OH_2_]^+^)^15^ in various crystalline systems,^16,17^ the PANO group features inherent chemical asymmetry, which makes the entire group interesting examples of benchmark systems. Experimental and computational investigations of the H-bond in PANO compounds in various phases unequivocally support the existence of a flat, asymmetric single-well proton potential with extremely complex proton dynamics involving anharmonicity, nuclear quantum effects, and strong coupling with the environment.^18−24^ While unsubstituted PANO has been the most extensively studied member of the group, the analogs are particularly interesting because the H-bond characteristics appear to be substantially influenced by electronegativity of the substituents.^19^ The H-bond of the PANO family also appears to be sensitive to the nonbonding interactions present in the crystalline solid state. The fact that PANO and its analogs crystallize in a variety of structural arrangements also provides an opportunity to investigate the influence of nonbonding interactions on the H-bond geometry.^25^ While the H-bond of PANO is of an intramolecular type, there exist closely related, chemically and structurally similar examples of intermolecular H-bonded complexes formed by pyridine *N*-oxide and strong carboxylic (trihaloacetic) acids.^26,27^

The H-bond of unsubstituted PANO is extremely short, *R*_O···O_ = 2.425 Å.^7^ Our previous infrared spectroscopy study of the PANO series in solution supported by theoretical calculations also suggested that the H-bond of MPANO is even shorter because of the electronic effects of the strongly electropositive methoxy group.^19^ Together with the intriguing vibrational dynamics, this merits a thorough experimental and computational investigation.

We also utilized conventional infrared spectroscopy, which has proven to be extremely valuable for the study of strong H-bonds. The position, shape, and behavior of OH stretching and out-of-plane OH bending during H/D exchange contain information about the hydrogen position and shape of the potential energy surface. However, in the case of strong H-bonds, this information is strongly mixed with the internal modes of the compound. Moreover, the identification of bands that correspond to normal modes with major participation of the COH bending and OH stretching internal coordinates is in general far from trivial because of the complex coupling mechanism with C=O, C–O, and N–O stretch internal coordinates. Coupling between OH stretching and low-frequency modes of the ···H···O subsystem must also be considered.

## 2 Methods and Models

### 2.1 Preparation of Samples

MPANO was synthesized from picolinic acid *N*-oxide (Sigma) following the procedure of ref (28). The H-bond proton was exchanged with deuterium in solution using methanol-d1, from which MPANO-D was recrystallized.

### 2.2 Neutron Diffraction

A preliminary X-ray data collection was carried out at 293(2) K (Mo Kα) to determine the molecular structure of MPANO as a starting model for the neutron data refinement.

Crystals are triclinic; space group *P*1̅, *a* = 6.7308(4) Å, *b* = 7.7246(3) Å, *c* = 8.1381(5) Å, α = 94.883(3)°, β = 106.998(3)°, γ = 112.171(3)°, *V* = 365.48(4) Å^3^, and *Z* = 2. 2992 reflections were collected (1666 unique). The structure was solved by direct methods and refined by full-matrix least-squares using anisotropic displacement parameters (ADPs) for the non-H atoms (*R* = 0.039, *R*_w_ = 0.041).

Neutron diffraction data were collected on single crystals of h7-MPANO (3.0 × 1.6 × 1.1 mm^3^). Measurement was made on the four-circle diffractometer D9 at the Institut Laue-Langevin, Grenoble, at a wavelength of 0.840 Å in a 2 K four-circle cryo-refrigerator. The crystal was cooled (∼2 K/min) to 20 K. No significant changes in the crystal mosaic or splitting of peaks was observed during cooling. The space group *P*1̅ was confirmed at 20 K. Unit cell dimensions: *a* = 6.6248(4) Å, *b* = 7.6188(4) Å, *c* = 8.1038(4) Å, α = 94.184(3)°, β = 109.042(3)°, γ = 111.464(3)°, and *V* = 351.12(3) Å^3^.

A total of 2794 reflections were collected, yielding 2476 unique reflections (*R*_int_ = 0.0112). The starting structural model was based on the atomic coordinates of the heavy atoms from the X-ray structure, while all hydrogen atoms were located from difference Fourier maps. The structure was refined by full-matrix least-squares using anisotropic displacement parameters (ADPs) for all atoms (*R* = 0.0395, *R*_w_ = 0.0855, on observed reflections with *I* > 2σ (*I*), GoF = 1.186). More data collection and refinement parameters, extended crystallographic data and references, an extended list of bond lengths, angles, and torsion angles are given in the CIF file and the Supporting Information.

### 2.3 Inelastic Neutron Scattering Spectroscopy

Inelastic neutron scattering spectra were collected on approximately 1g of the material at 20 K on the inverse geometry time-of-flight spectrometer FDS of Manuel Lujan Jr., Neutron Scattering Center of Los Alamos National Laboratory. The samples were sealed under He in an aluminum can, which was mounted in a closed-cycle refrigerator placed on the spectrometer. Data were reduced and treated with instrument-specific analysis programs.^29^

### 2.4 Infrared Spectroscopy

Samples were prepared as KBr pellets and recorded in transmission mode on a Bruker Vertex 70 spectrometer. For low-temperature measurements, a Harrick low-temperature cell, purged with dry nitrogen, was applied. A homemade controller was used to control and monitor the temperature in the range between room temperature and −150 °C. Typically, 256 scans were averaged with a nominal resolution of 2 cm^–1^. The deuterated samples were prepared as described above.

### 2.5 DFT Calculations

All calculations reported herein were performed with the program package VASP v. 5.3.5.^30−33^ The program employs quantum treatment of the electronic structure based on density functional theory with periodic boundary conditions implemented. We used the Perdew–Burke–Ernzerhof (PBE) functional^34^ corrected for dispersion effects by the DFT-D3 method of Grimme,^35^ together with the Projector Augmented Wave ultrasoft pseudopotentials^36,37^ and a plane wave basis set with a cutoff of 500 eV. Electronic integrals in the reciprocal space were computed on a 4 × 4 × 4 Monkhorst–Pack mesh of *k*-points.^38^ The energy-stopping criterion for the electronic relaxation was set to 10^–6^ eV.

Starting from the experimental crystal structure acquired by ND (see above), the entire system (atomic positions and unit cell parameters) was optimized by rigorously following symmetry constraints of the *P*1̅ space group using a force-stopping convergence criterion of 0.003 eV/Å. Optimization was followed by harmonic frequency calculation in which the Hessian matrix was computed by the finite difference method with atomic displacements of 0.015 Å and taking into account symmetry features of the pertinent space group.

In addition to periodic calculations, a model of isolated MPANO was constructed and treated with the same approach as described above by placing a single molecule into a large cubic cell of 20 Å to diminish intermolecular interactions (note that such calculation remains formally periodical). All of the calculation settings remained the same, except that the unit cell parameters were kept fixed and electronic integrals were computed only at the Γ-point.

In order to further elucidate the selected characteristics of MPANO, another isolated model was created and treated at the M06-2X/6-31+G(d,p) level of theory. The electronic structure of an optimized MPANO molecule was analyzed by the natural bond orbital (NBO) methodology,^39^ implemented in the NBO v. 7.0.4 program,^40^ focusing on the interaction between orbitals in the H-bond moiety. Following that, the same analysis was repeated on the PANO molecule obtained by replacing the methoxy group of MPANO with hydrogen and keeping the rest of the structure in the same geometry. Additionally, anharmonic frequencies were computed for the optimized MPANO molecule by numerical differentiation along normal modes. All these calculations were carried out by the Gaussian 16 program.^41^

The INS spectra were computed from the amplitudes and frequencies of the calculated normal modes by using the a-Climax v. 5.5.0 program,^42^ including overtones and phonon wings in the calculation of the INS intensity. Assignment of the computed INS spectra was facilitated by visualization of the respective normal modes using the Jmol utility.^43^

## 3 Results and Discussion

### 3.1 Crystal Structure and H-Bond Geometry—Neutron Diffraction

**Figure 1:**
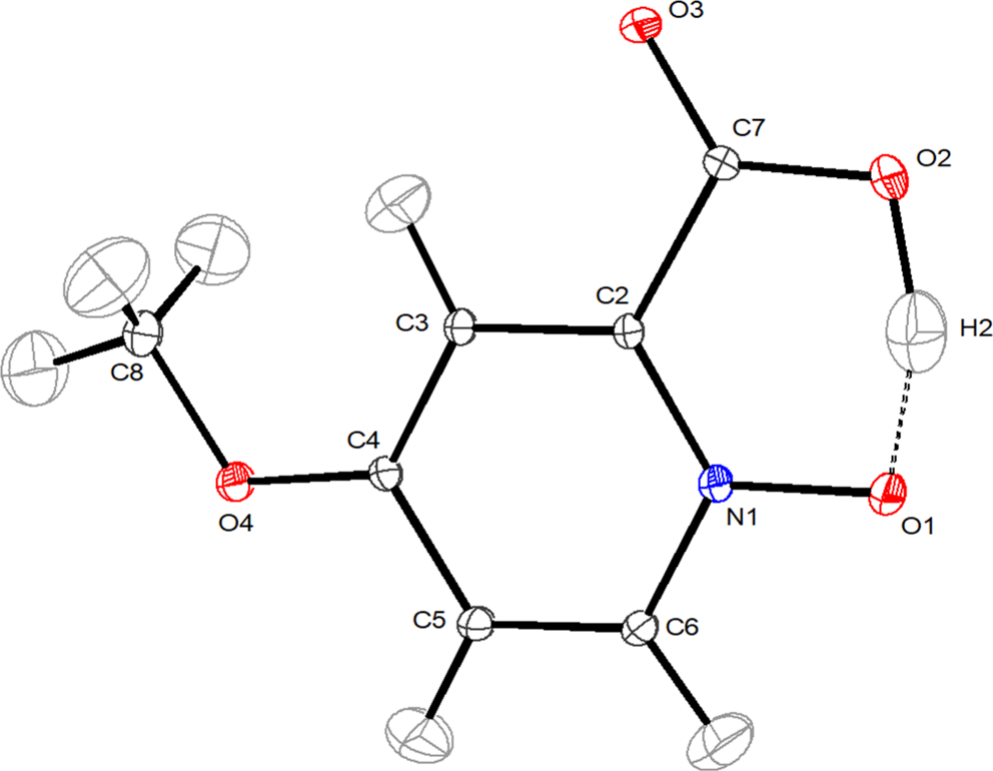
Ortep view of the MPANO molecule. (20 K, thermal ellipsoid drawn at 70% probability).

An Ortep view of the MPANO molecule is shown in Figure 1, while relevant bond lengths and angles together with the corresponding calculated values are listed in Table 1. Measured and computed unit cell parameters are listed in Table 2. Extended crystallographic data are listed in the SI, Tables S1–S6.

**Table 1:** Selected Interatomic Distances (Å) and Angles (deg) for MPANO Measured by Neutron Diffraction (ND) and Computed by the Isolated and Periodic Model

	ND	isolated model	periodic model
O1–H2	1.271(2)	1.479	1.372
O2–H2	1.171(2)	1.048	1.088
O1–O2	2.403(1)	2.478	2.419
O1–N1	1.349(1)	1.322	1.336
O2–C7	1.295(1)	1.329	1.318
O3–C7	1.219(1)	1.224	1.230
O4–C4	1.333(1)	1.354	1.341
O4–C8	1.438(1)	1.437	1.441
O1–H2–O2	159.3(2)	157.0	159.0
O2–C7–C2	116.08(7)	115.59	116.10
C7–O2–H2	104.3(1)	105.8	104.5
N1–O1–H2	99.8(1)	98.7	99.3

**Table 2:** Measured (ND) and Optimized Unit Cell Parameters of MPANO^a^

	ND	calculation (periodic model)
*a* [Å]	6.6248(4)	6.6454
*b* [Å]	7.6188(4)	7.6398
*c* [Å]	8.1038(4)	8.1213
α [deg]	94.184(3)	94.40
β [deg]	109.042(3)	108.60
γ [deg]	111.464(3)	111.41
*V* [Å^3^]	351.12(3)	354.98

a All lengths are given in Å and angles are given in deg.

The observed geometrical parameters in MPANO are comparable to those found in similar molecules such as unsubstituted PANO^7,23^ and its quinoline-based analog quinaldic acid *N*-oxide (QANO)^7^ as well as 4-nitropicolinic acid *N*-oxide (NPANO).^44^ For example, the N1–O1 separation at 1.349(2) Å is longer than that found in NPANO at 1.323(2) Å and in QANO at 1.332(2) Å but comparable with that in PANO at 1.342(2) Å. The C7–O2 and C7–O3 distances at 1.295(1) and 1.219(1) Å are not significantly different from the analogous distances in the above-mentioned compounds at 1.305(3), 1.305(2), and 1.309(2) Å. Moreover, other distances are also similar and in the expected range. The small but hardly significant differences can be attributed to electronic effects, mainly of the functional group bound to C4, as well as to different packing environments in the three compounds.

The most significant feature in the MPANO structure is the asymmetric intramolecular H-bond O2-H2···O1 with a very short oxygen–oxygen separation of 2.403(1) Å. The D–H bond is elongated, and correspondingly, the H···A distance is shortened (O2–H2 1.171(2) Å and H2–O1 1.271(2) Å, respectively) as was previously observed^45^ in the case of strong H-bonds (in the range of 2.4–2.5 Å) Moreover, the anisotropic displacement parameter of the H2 atom shows a large amplitude motion along the O1–O2 direction. The O2–H2–O1 angle is 159.3(2)°, possibly because of stereochemical restrictions. These geometrical parameters may be compared with those in similar H-bonding patterns found in the X-ray structures of NPANO (O···O 2.460(2) Å and 159(2)°), PANO (2.425(2) Å and 153(2)°), and ammonium hydrogen maleate (2.432(2) Å and 174(4)°).^12^ In another single-crystal neutron diffraction structure, that of hydrogen phthalate at 30 K,^11^ the O···O separation is 2.528(8) Å and the D···H and H···A distances are 1.075(15) Å and 1.456(16) Å, respectively. The O–H···O angle is 175(1)°. The H-bond of MPANO is enhanced possibly because of the strong electron-donating effect of the methoxy group at the ring position, which makes it the shortest among the members of the PANO family and also among the shortest known O–H···O bonds, while the longer H-bond in NPANO may be the result of the nitro functional group with an opposite electronic effect.

The MPANO unit cell content is shown in the SI, Figures S1 and S2, while molecular packing is displayed in Figures S3 and S4 and selected packing distances are shown in Figures S5 and S6. Molecules form two-dimensional (2D) sheets, perpendicular to the crystallographic *a* axis, in which each molecule is interacting to the others via C–H···O–N and C–H···O=C interactions (at 2.14 and 2.17 Å, respectively) and weaker ones at 2.61 Å (Figure S6). The 2D layers are held together by C···H···O contacts (at ca. 2.4 and 2.7 Å) and π–π interactions (Figure S5). The interlayer distance is 3.3 Å. Hirshfeld surfaces are displayed in the SI, Figures S7–S10. Details of the D···H···A interactions for MPANO are shown as 2D fingerprint plots^46,47^ in Figure S11, which represent the overall, H···O, H···N, H···C, and C···O contacts. Electrostatic potential is displayed in Figure S12.

#### 3.1.1 Crystal Structure and H-Bond Geometry—Calculations

The periodic DFT optimization was able to reproduce the measured unit cell parameters very accurately. While the calculation predicts the cell constants to be slightly larger (Table 2), the relative error is not more than about 0.3%, and the calculated volume is ∼1% larger than that measured. The arrangement of PANO molecules in the crystal structure appears to be in an excellent match with the ND structure (an overlay of experimental and calculated unit cells demonstrating the match is displayed in the SI, Figure S13). Both the internal geometry of MPANO and the arrangement of molecules in the crystal structure are nearly identical in the experiment and computation, which is reflected in the fact that the RMSD of atomic positions (including hydrogen atoms) between the computed and measured structure amounts to only 0.060 Å; these offsets are nearly imperceptible on the overlay (Figure S13). Note that much larger offsets, up to 0.25 Å (and typically excluding hydrogen atoms), are accepted to declare the calculation as accurate.^23^ The largest mismatch between the optimized and ND structures is the location of the proton in the H-bond, where the difference amounts to 0.109 Å. Apparently, the calculation predicts the proton location to be noticeably closer to the donor oxygen atom (Table 1). The differences in the geometry of the H-bond and its close surroundings can be further examined by comparing the most relevant interatomic distances listed in Table 1. Here, geometric parameters of the gas-phase optimization are also included.

The gas-phase calculation underestimates the shortness of the H-bond to a considerable degree, as it finds an optimized O···O distance, which is 0.075 Å longer than that measured and the location of the proton firmly at the donor site in contrast to the periodic model, which yields much better agreement. Other bond length characteristics of the H-bond are less affected by the type of model and generally differ from the measured values by no more than 0.02 Å, which can be considered to be very good agreement. This comparison suggests that the H-bond of MPANO is substantially influenced, in the present case enhanced, by nonbonding interactions established by crystal packing, despite the fact that the H-bond is of intramolecular nature. The same observation was made for the unsubstituted PANO^23,25^ with a rather different arrangement of molecules in the crystal structure.^7^ We note that despite the good agreement of the periodic calculation for the H-bond geometry, the O···O separation is still slightly overestimated (by 0.016 Å) and that the proton is located 0.083 Å nearer the donor oxygen. This disagreement can readily be attributed to nuclear quantum effects on account of the pronounced quantum nature of the hydrogen nucleus in extremely short H-bonds. As the proton wave function tends to penetrate toward the midpoint of an H-bond, the coupling with the donor···acceptor internal degree of freedom results in slight shortening of the donor···acceptor separation and an elongation of the O–H distance.^16,19^ These effects are, of course, reflected in the actual structure but require advanced computational treatments.^16,19^ We have recently been able to elucidate nuclear quantum effects in the example of unsubstituted PANO in a crystalline solid by using nuclear quadrupole resonance spectroscopy in conjunction with periodic DFT calculations and quantization of nuclear motion along selected internal coordinates. This resulted in a superior match with the H-bond geometry determined by ND.^24^ While such a detailed approach is beyond the scope of the present work, it can be safely deduced that inclusion of nuclear quantum effects would result in further improvement of the already good agreement between the periodic calculation and ND results. In summary, discrepancy between the routinely computed geometry in the gas phase and experimental geometry in the crystal can be attributed to the (i) lack of interactions present in the crystal field and (ii) lack of nuclear quantum effects.

The role of orbital interactions in the shortness of the H-bond of MPANO has been demonstrated by NBO analysis for the optimized isolated MPANO molecule, as well as for an unsubstituted PANO molecule. A similar approach was also used in the past to elucidate the role of the cooperative effect in the short H-bonds in crystalline oxalic acid dihydrate.^48^ In order to ensure comparability, all common structural motifs were kept in the same geometry in both the MPANO and PANO molecules. We observed a strong H-bond enhancing tendency through the stabilizing interaction between the electron lone pair on the acceptor (N-oxide) oxygen atom and the O–H antibond orbital. This interaction is schematically displayed in Figure S14. The lone pair, an electron donor orbital, pushes some electron density into the antibonding orbital, thereby weakening the O–H bond and enhancing the H···O interaction accompanied by the shortening of the O···O distance. MPANO exhibits a stronger H-bond enhancement effect than PANO. For MPANO, the stabilization interaction between the lone pairs of the acceptor (N-oxide) oxygen and the O–H antibond amounts to 71.2 kcal/mol, whereas for unsubstituted PANO frozen in the same geometry, this interaction is slightly weaker, at 70.1 kcal/mol. At the same time, the energy of the O–H bonding orbital of MPANO amounts to −0.768 au, making it less stable than the same orbital of PANO at −0.772 au. These differences are solely due to the influence of the methoxy group at the p-position and indicate a stronger H-bond shortening tendency in MPANO.

We can hereby confirm that MPANO possesses one of the shortest O–H···O bonds known. With an O···O separation of 2.403(1) Å, it is matched or surpassed in shortness only by few systems such as hydrogen maleate (see ref (12)) or hydrogen phthalate (see ref (11)). Moreover, this is a rare example of intrinsically asymmetric H-bonding of such a shortness. The proton is positioned 1.171(2) Å from the donor and 1.271(2) Å from the acceptor oxygen atom, which reflects the asymmetry of the single well-type potential.

### 3.2 Vibrational Spectra

**Figure 2:**
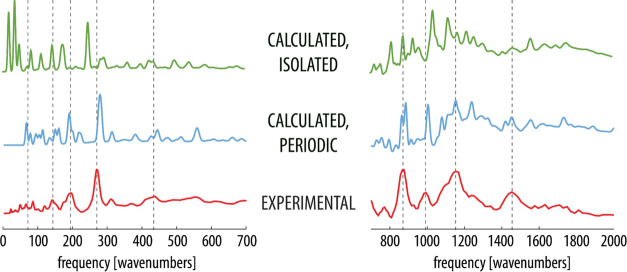
Experimental and computed (with the isolated and periodic model) INS spectra of normal MPANO. Dashed vertical lines correspond to the peaks of selected bands in the experimental spectrum for easier comparison. Note that the *y*-axis of the plots corresponds to the INS intensity given in arbitrary units.

**Figure 3:**
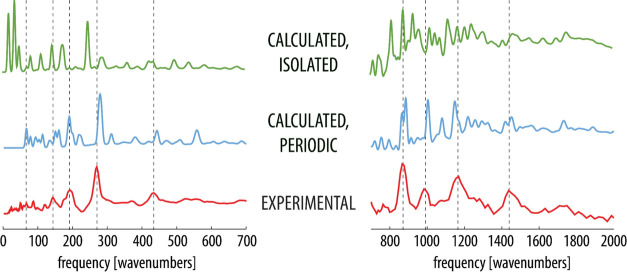
Experimental and computed (with isolated and periodic model) INS spectra of H-bond-deuterated MPANO. Dashed vertical lines correspond to the peaks of selected bands in the experimental spectrum for easier comparison. Note that the *y*-axis of the plots corresponds to INS intensity given in arbitrary units.

The vibrational spectra collected by INS and IR spectroscopies on both normal and H-bond-deuterated MPANO serve to highlight the special dynamics of this very short H-bond, which in the case of INS are supported by quantum calculations. The computed and measured spectra of normal and H-bond-deuterated MPANO are displayed in Figures 2 and 3, respectively. Note that for ease of presentation, the spectra are split into two frequency ranges from 0 to 700 cm^–1^ and from 700 to 2000 cm^–1^ since the spectra are largely featureless at higher frequencies. Here, we mainly focus on the spectral features related to the H-bond, but a more thorough assignment of the computed spectra can be found in the SI, Figure S15.

A general comparison reveals that for both isotopomers, the periodic model reproduces the position and shape of bands in the experimental spectra noticeably better than the isolated molecule model. This is apparent in practically all the most pronounced bands and confirms that periodicity in the computational treatment is essential in order to correctly reproduce the observables as did the significantly better match of the optimized geometric parameters.

**Figure 4:**
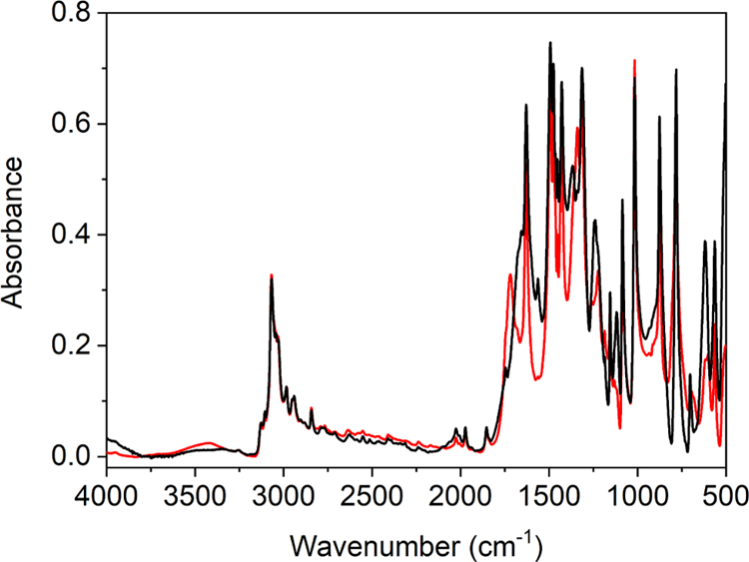
Infrared spectra of protic (black) and deuterated (red) MPANO recorded at *T* = 21 °C.

Assignment of the MPANO IR vibrational spectra in normal and H-bond-deuterated forms is partially based on assignments in PANO.^19,21^ The shorter O···O spacing and the associated stronger intramolecular H-bonding in the MPANO molecule should give rise to some changes in the bands most sensitive to the hydrogen bonding properties. The IR spectra of MPANO in normal and deuterated forms are presented in Figure 4, and the fingerprint region of both forms is displayed in the SI, Figure S16.

Perhaps, the most important aspect of the IR spectra is the expected very broad O–H stretching band, as it has no counterpart in the INS spectra. Its precise position is rather difficult to determine, as, for example, in PANO, this continuum extends from the carbonyl bands to the far-infrared region.^21^ A reliable quantitative characterization of the continuum in the MPANO spectrum in terms of the position of the band maximum and the band shape is, however, not possible because of the numerous overlapping peaks and Evans transmissions. The center of gravity also cannot be determined for the same reason. A curve fitting procedure did locate the position of the broad band maximum near 1360 cm^–1^, which is lower than in the spectrum of PANO. This is consistent with the shorter H-bond found in crystalline MPANO. The O–H stretching band, however, extends toward 500 cm^–1^ as in the case of PANO (see the SI, Figure S17), and is indicated by the presence of multiple Evans transmissions. While the high-frequency part of the O–H stretching band was modified by H/D exchange in the PANO spectrum,^19,21^ no significant effect of deuteration was observed in the O–H stretching band of MPANO.

A comparison of the IR spectra of PANO and MPANO in the fingerprint region (SI, Figure S16) makes it clear that the “continuum” of the O–H stretching band has a very different shape. While in the case of PANO, we speak of a broad O–H stretching mode with several well-defined Evans transmissions extending monotonically throughout the fingerprint region, the shape of the O–H stretching in MPANO is quite different. It appears to have a broad minimum around 1100 cm^–1^ that is very difficult to assign because of the presence of Evans transmissions. The shape of the O–H stretching band also has a more complex structure than in the case of PANO. Lowering the temperature has no observable effect on the spectra other than the expected narrowing of the internal vibrational bands and small shifts in the frequencies. This is true for both the normal and deuterated forms of MPANO (see the SI, Figures S18 and S19).

Neither the experimental nor computed INS spectra, however, show any clear evidence for the O–H/O–D stretching mode, which can possibly be rationalized by coupling with other modes, which has the effect of smearing the intensity for this mode over a wide frequency range, thereby diminishing its intensity. Indeed, inspection of the hydrogen displacements for the corresponding computed modes reveals that the hydrogen displacements are quite small. Since the stretching band is, however, clearly visible in the infrared spectra (Figure 4), the validity of our calculations can be at least qualitatively determined. The accuracy of the computed harmonic frequencies in the case of the O–H/O–D stretching mode in short H-bonds is generally questionable, since the stretching mode exhibits a high degree of anharmonicity, is often red-shifted, and is extensively (anharmonically) coupled with other vibrations. Our gas-phase calculation predicts the O–H stretching mode at 2452 cm^–1^ for the H-isotopomer, whereas the periodic calculation finds it at just over 2000 cm^–1^. These modes also include a visible contribution of stretching of the C=O group. Similarly, the OD stretching component for the D-isotopomer appears in two modes at 1819 and 1707 cm^–1^ in the gas phase, but in the crystal, the frequencies are lower, in the range between 1700 and 1430 cm^–1^. The O–D stretching contribution in most of these modes is weak with the prevailing contribution of C=O stretching, N–O stretching, and in-plane ring deformation modes (see the SI, Figure S15). All the computed stretching frequencies appear to be too high in comparison with the experimental infrared spectra, often by hundreds of wavenumbers. These discrepancies clearly reflect the limitations of the harmonic approximation. The present evaluation of anharmonic frequencies for an isolated MPANO molecule confirms this: while the harmonic O–H stretching frequency computed at the M06-2X/6-31+G(d,p) level amounts to 2644 cm^–1^, it shifts to 1951 cm^–1^ due to anharmonicity. The red shift of nearly 700 cm^–1^ indicates an extraordinarily strong and anharmonic H-bond. All other modes feature a several times smaller anharmonicity effect at best. Because of the neglection of crystal lattice effects, the computed anharmonic O–H stretching frequency is still evidently overestimated.

While an equivalent anharmonic treatment could not be imposed on the periodic model, we believe that it would have a significant impact on the coupling scheme between the modes. Namely, the harmonic O–H stretching mode includes perceivable contribution of other vibrations and at the same time, the O–H stretching motion can be detected in several other modes; still, its frequency of 2007 cm^–1^ is probably too high to reveal the entire coupling network—virtually, no other mode shows up at that frequency, but the frequency match is definitely required for extensive coupling to occur. For the O–D stretching mode however, this is quite different, in that the harmonic frequency of <1700 cm^–1^ falls in the range abundant with other modes. Consequently, the O–D stretching component is present in a number of different modes in the range between 1700 and 1430 cm^–1^. Notably, there is nothing like a “pure” O–D stretching mode; rather than that, the O–D contribution (usually minor) appears in several modes over a wide frequency range. Similarly, the enormous downshift of the O–H stretching frequency (nearly 700 cm^–1^) suggests that in the crystal, the computed anharmonic mode would be shifted to 1300–1400 cm^–1^, i.e., into the range abundant with bending and ring modes. While we are unable to devise any quantitative coupling scheme for such a scenario, we argue that the O–H stretching mode would be heavily smeared over a number of modes in a wide frequency range, just as is the presently computed O–D mode, but likely to a larger extent.

Although the intriguing feature of the “invisible” O–H stretching mode in the INS spectrum cannot be quantitatively elucidated by calculations due to limitations inherent to harmonic approximation, the present calculations may be viewed as correct in the sense that they suggest significant coupling with other modes.

In contrast to H-bond modes, calculations provide a sound and quantitative agreement for characteristic vibrations of the methyl group, namely, CH_3_ libration and bending modes. Periodic calculations quite accurately predict the libration mode to be at 278 cm^–1^, as compared to the experimental INS band at 270 cm^–1^. The isolated model is much less accurate, yielding an underestimated frequency of 248 cm^–1^. Explanation of this discrepancy is trivial: the isolated molecules lack CH_3_...O contacts established in the crystal, anchoring the methyl groups and stiffening the corresponding force constants. Consequently, the isolated model predicts considerably lower methyl libration frequency. In contrast to that, both isolated and periodic calculations predict the CH_3_ bending modes to have very similar frequencies, in the range between 1409 and 1467 cm^–1^ (isolated model) and 1417 and 1483 cm^–1^ (periodic model); in all cases, the bending vibrations are mixed with ring vibrations. The agreement of the estimated CH_3_ bending modes is in good agreement with the out-of-phase bending vibration in the IR spectra located at 1453 cm^–1^ but somewhat less so with the in-phase CH_3_ bending mode; however, note that the assignment of the latter to the IR peak at 1340 cm^–1^ is less certain. Visual inspection of the methyl bending modes suggests that in the crystal, the C···H···O contacts are somewhat less disrupted by the hydrogen motion than in the case of methyl libration, which explains the perceivably smaller difference between the isolated and periodic calculation.

The low-frequency region of the INS spectrum for the H-isotopomer consists of many bands resulting from very complex vibrations of MPANO molecules, and most of these bands include a donor···acceptor component (i.e., O···O motion). Modes with significant O···O contribution appear at 190 cm^–1^ and extend to 730 cm^–1^, in good agreement with peaks in the experimental spectrum (see the SI, Figure S15). The presence of the donor···acceptor stretching component in such a broad frequency range may be indicative of a strong H-bond in which the donor···acceptor motion is substantially coupled with a number of low-frequency internal modes. Very similar assignments can be also devised for the D-isotopomer (Figure 3 and SI, Figure S15).

The high-frequency region is quite complex, but differences between normal MPANO and H-bond-deuterated MPANO help identify signatures of modes related to the H-bond and its neighbors. A general feature of the INS spectra of MPANO is its relatively low sensitivity to deuteration. Perhaps, the largest difference between isotopomers can be observed in the broad, structured band between 1050 and 1350 cm^–1^ with a maximum at about 1160 cm^–1^. Our calculations suggest that this maximum may be attributed to the HCH and OCH bending of the methoxy group for both isotopomers (SI, Figure S15). In the case of the H-isotopomer, this band exhibits a pronounced shoulder at ∼1100 cm^–1^, which disappears on deuteration. It can therefore be assigned to the out-of-plane bending of the H-bonded proton. According to our calculations, this band shifts to about 800 cm^–1^ upon H-bond deuteration with substantial loss of intensity (as expected due to the much lower scattering power of D relative to H) and is well matched with a weak band between 750 and 800 cm^–1^ in the experimental spectrum (SI, Figure S16). This is in agreement with the IR spectra where a comparison between the normal and H-bond deuterated form suggests only one candidate for the out-of-plane O–H bending, located at 1115 cm^–1^ (Figure 4 and SI, Figure S16), which is indicative of the shortness of the intramolecular H-bond. The IR spectrum at low temperature shows some intrinsic band structure from the reduction in halfwidths and suggests that the band at 817 cm^–1^ can be assigned to the out-of-plane O–D bending mode.

The right shoulder of the INS methoxy bending peak at ∼1250–1300 cm^–1^ shows little difference between isotopomers and, according to calculations, corresponds to the in-plane ring deformation modes as well as C–O and N–O stretching vibrations near the H-bond (SI, Figure S15). For the D-isotopomer, some of these modes include a significant in-plane O–D bending component. The last sizable peak is located at ∼1450 cm^–1^. It is broad but barely affected by deuteration and consists of in-plane ring deformation modes accompanied by in-plane COH (COD) bending and HCH/OCH bending of the methoxy group. In addition, for the D-isotopomer, this peak includes a weak O–D stretching component. Above 1700 cm^–1^, all the features become weak and are interpreted as overtones or combinations of vibrational transitions.

The carboxylate region between 1750 and 1550 cm^–1^ in the IR spectrum (Figure 4 and Si, Figure S16) is composed of bands originating from the carboxyl and ring moiety of the compound. The frequency of the ring vibration should be insensitive to deuteration. Therefore, we assign the most intense band in this region at 1629 cm^–1^ and the shoulder at about 1673 and 1745 cm^–1^ to the coupled C–C and overtone ring modes, respectively. However, the band at 1656 cm^–1^ is sensitive to H/D exchange as it shifts upon deuteration to 1718 cm^–1^. This band is assigned to the mode with a predominant C=O stretching character. It is likely coupled with the H-bond stretching and bending modes according to our calculations, which is in agreement with the observed sensitivity to deuteration.

## 4 Conclusions

Examples of extremely short H-bonds are rare and not well understood, despite extensive research efforts devoted to H-bonding in general. The reason for this is that only a limited number of examples of such H-bonds are known and that their characterization most often presents an exceedingly difficult task because of the highly complex H-bond dynamics. Unlike the more conventional experimental methods, neutron techniques facilitate a different and to a certain extent complementary insight into extremely short H-bonding. For instance, single-crystal neutron diffraction can accurately locate the position of the H atom, while inelastic neutron scattering spectroscopy is capable of detecting certain vibrational modes not readily accessible to optical vibrational spectroscopy.

Our neutron diffraction studies confirm the assumption that the H-bond of MPANO is among the very shortest known; in addition, it is evidently asymmetric unlike most of the systems with a similar, short H-bond. The structure determined by diffraction has been validated by quantum calculations in excellent agreement both for the unit cell parameters and for the internal geometry of MPANO molecules. A minor exception to this is the slight deviation of the hydrogen atom location (underestimated O–H and overestimated O···H distance). This can readily be explained by nuclear quantum effects, which is beyond the scope of this work, but has been reported to improve the calculation.^24^ Finally, a comparison between gas-phase and periodic calculation suggests that nonbonding interactions from the crystal field significantly contribute to shortness of the H-bond, an effect previously observed for unsubstituted PANO.^23,25^ An in-depth analysis of the influence of the nonbonding interactions in the crystal packing represents a challenge but requires complex treatments, e.g., calculations on clusters of neighboring molecules, periodic calculations in reduced dimensionality (2D, 1D), and so forth. Because many of these interactions are relatively weak, the limited precision of DFT may not be sufficient for the proper assessment of factors governing the structure of individual molecules, let alone the crystal structure.

INS and IR spectroscopies yield somewhat complementary information about H-bond dynamics. The most prominent feature of the IR spectrum of MPANO is the intense, broad, and complex band spanning approximately between 1800 and 500 cm^–1^ similar to other systems with short H-bonds. This band is topped by several other bands and cut in several places by Evans transmissions. It is assigned to the O–H (O–D) stretching mode; its shape, breadth, and position are indicative of an extremely short H-bond with prominent anharmonicity and coupling with other H-bond vibrations. While the harmonic frequency calculation obviously cannot properly reproduce these features, it does at least indicate heavy mixing between H-bond vibrations. The O–H stretching mode is not detectable in the INS spectra in contrast to IR, and this is consistent with extensive coupling to a number of other modes over a wide frequency range. The other clearly detectable H-bond mode is the out-of-plane O–H (O–D) bend, which appears at about 1100 (H) and 800 cm^–1^ (D) in both INS and IR spectra and is neatly reproduced by our calculation. The relatively high frequency of this mode is further indicative of the shortness and strength of the H-bond.

MPANO in conjunction with other members of the PANO family (Scheme 1) represents an intriguing case of very short, asymmetric intramolecular H-bonds suitable for in-depth investigation of factors governing the H-bond shortness, among electronic structure effects of the substituents and effects of the crystalline environment. PANO with its well-studied analogs is one of the best benchmark systems of short H-bonding, possibly suitable for further unveiling the enigmatic nature of this peculiar interaction.

## Supporting Information Available

Atomic parameters, isotropic and anisotropic displacement parameters (ADPs), bond lengths, angles, lsq-planes, Orteps, packing diagrams, H-bond interactions, and Hirshfeld plots for MPANO (PDF); comparison between computed and measured crystal structure of MPANO; details on NBO electronic structure analysis; detailed assignment of INS spectra assisted by calculations; comparison between MPANO/PANO infrared spectra recorded at different conditions.
Crystallographic data (CIF)

## Supplementary Material

ao4c05344_si_001.pdf

ao4c05344_si_002.cif
