# Supplementary material for: 4-Methoxypicolinic Acid N-Oxide: One of the Shortest Hydrogen Bonds Known Characterized by Neutron Diffraction, Inelastic Neutron Scattering, Infrared Spectroscopy, and Periodic DFT Calculations
Source: ACS Omega. 2024 Aug 29;9(36):38116–25. doi: 10.1021/acsomega.4c05344 (PMC11391546; doi:10.1021/acsomega.4c05344)
Supplement: Supplementary file 1 — ao4c05344_si_001.pdf [file ao4c05344_si_001.pdf]

4-methoxy picolinic acid *N*-oxide: one of the shortest hydrogen bonds known, characterized by neutron diffraction, inelastic neutron scattering, infrared spectroscopy and periodic DFT calculations

SUPPLEMENTARY INFORMATION

Jernej Stare,[a,] Jože Grdadolnik,[a] Sax Mason,[b] Alberto Albinati,[c] Juergen Eckert [d,]**

[a] *Theory Department, National Institute of Chemistry, Hajdrihova 19, SI-1000 Ljubljana, Slovenia*
[b] *Institut Laue-Langevin, 6 rue Jules Horowitz, BP 156, 38042 Grenoble Cedex 9, France*
[c] *CNR-ICCOM, Via Madonna del Piano, 50119 Sesto Fiorentino, and University of Milano, Milano, Italy*
[d] *Department of Chemistry and Biochemistry, Texas Tech University, P. O. Box 41061, Lubbock, TX 79409-1061, USA*

** Corresponding authors, e-mail: jernej.stare@ki.si / juergen.eckert@proton.me*

Table S1. Crystal data and structure refinement for MPANO.

Empirical formula	$C_7H_7NO_4$	
Formula weight	169.14	
Temperature, K	20(2)	
Wavelength, Å	0.8404	
Crystal system	Triclinic	
Space group	*P*-1	
Unit cell dimensions	*a* = 6.6248(4) Å	*α*= 94.184(3) deg.
	b = 7.6188(4) Å	*β*= 109.042(3) deg.
	c = 8.1038(4) Å	*γ* = 111.464(3) deg.
Volume, $Å^3$	351.12(3)	
Z	2	
D_{calc}, Mg/m^3	1.600	
Absorption coefficient, mm^{-1}	0.173	
F(000)	106	
Crystal size, mm	3.0 x 1.6 x 1.1	
θ range for data collection, deg	4.20 to 40.01	
Index ranges	-10<=h<=2, -10<=k<=11, -10<=l<=12	
Reflections collected	2794	
Independent reflections	2476 [R_{int} = 0.0112]	
Completeness to θ = 40.01 deg	93.8 %	
Absorption correction	None	
Refinement method	Full-matrix least-squares on F^2	
Data / restraints / parameters	2476 / 0 / 173	
Goodness-of-fit on F^2	1.186	
Final R indices [I>2σ(I)]	R_1 = 0.0395, wR^2 = 0.0855	
R indices (all data)	R_1 = 0.0452, wR^2 = 0.0881	
Extinction coefficient	0.027(2)	
Largest diff. peak and hole	0.768 and -0.844 fm $Å^{-3}$	

Table S2. Atomic coordinates (x 10^4) and equivalent isotropic displacement parameters ($Å^2$x 10^3) for MPANO. U(eq) is defined as one third of the trace of the orthogonalized U^{ij} tensor.

	x	y	z	U(eq)
O(1)	516(2)	971(1)	-2530(1)	8(1)
O(2)	1004(2)	2317(1)	422(1)	8(1)
O(3)	2624(2)	5524(1)	1415(1)	8(1)
O(4)	4179(2)	8089(1)	-3904(1)	8(1)
N(1)	1430(1)	2769(1)	-2805(1)	6(1)
C(2)	2120(1)	4345(1)	-1522(1)	5(1)
C(3)	3057(1)	6183(1)	-1831(1)	6(1)
C(4)	3300(1)	6402(1)	-3460(1)	6(1)
C(5)	2555(1)	4736(1)	-4759(1)	6(1)
C(6)	1639(1)	2942(1)	-4400(1)	7(1)
C(7)	1910(1)	4084(1)	259(1)	6(1)
C(8)	5023(2)	9832(1)	-2592(1)	9(1)

Table S3. H atoms coordinates (x10^4) and isotropic displacement parameters ($Å^2$x10^3) for MPANO.

	x	y	z	U(eq)
H(2)	609(3)	1381(3)	-950(3)	21(1)
H(3)	3565(4)	7386(3)	-755(3)	21(1)
H(5)	2665(4)	4864(3)	-6058(2)	20(1)
H(6)	1021(4)	1585(3)	-5337(3)	21(1)
H(8A)	3576(4)	9926(3)	-2268(3)	26(1)
H(8B)	5709(5)	11009(3)	-3204(3)	28(1)
H(8C)	6422(4)	9875(3)	-1396(3)	27(1)

Table S4. Bond lengths [Å] and angles [°] for MPANO.

O(1)-N(1)	1.3486(10)
O(1)-H(2)	1.271(2)
O(2)-C(7)	1.2950(11)
O(2)-H(2)	1.171(2)
O(3)-C(7)	1.2192(12)
O(4)-C(4)	1.3334(11)
O(4)-C(8)	1.4382(12)
N(1)-C(6)	1.3546(9)
N(1)-C(2)	1.3553(9)
C(2)-C(3)	1.3876(10)
C(2)-C(7)	1.5145(10)
C(3)-C(4)	1.3955(10)
C(3)-H(3)	1.086(2)
C(4)-C(5)	1.4034(11)
C(5)-C(6)	1.3739(10)
C(5)-H(5)	1.0876(19)
C(6)-H(6)	1.089(2)
C(8)-H(8A)	1.098(2)
C(8)-H(8B)	1.081(2)
C(8)-H(8C)	1.089(2)
N(1)-O(1)-H(2)	99.80(11)
C(7)-O(2)-H(2)	104.32(11)
C(4)-O(4)-C(8)	118.11(8)
O(1)-N(1)-C(6)	117.74(6)
O(1)-N(1)-C(2)	120.85(6)
C(6)-N(1)-C(2)	121.41(6)
N(1)-C(2)-C(3)	119.98(7)
N(1)-C(2)-C(7)	119.59(6)
C(3)-C(2)-C(7)	120.42(7)
C(2)-C(3)-C(4)	119.79(7)
C(2)-C(3)-H(3)	116.53(13)
C(4)-C(3)-H(3)	123.68(13)

O(4)-C(4)-C(3)	125.10(7)
O(4)-C(4)-C(5)	116.32(7)
C(3)-C(4)-C(5)	118.58(7)
C(6)-C(5)-C(4)	119.84(7)
C(6)-C(5)-H(5)	120.01(13)
C(4)-C(5)-H(5)	120.13(13)
N(1)-C(6)-C(5)	120.41(7)
N(1)-C(6)-H(6)	115.35(13)
C(5)-C(6)-H(6)	124.24(13)
O(3)-C(7)-O(2)	125.59(8)
O(3)-C(7)-C(2)	118.32(7)
O(2)-C(7)-C(2)	116.08(7)
O(4)-C(8)-H(8A)	110.10(14)
O(4)-C(8)-H(8B)	105.86(14)
H(8A)-C(8)-H(8B)	110.1(2)
O(4)-C(8)-H(8C)	110.18(14)
H(8A)-C(8)-H(8C)	110.9(2)
H(8B)-C(8)-H(8C)	109.6(2)

Table S5. Anisotropic displacement parameters ($Å^2$x 10^3) for MPANO. The anisotropic displacement factor exponent takes the form: $-2\pi^2[h^2a^{*2}U^{11} + ... + 2 h k a^* b^* U^{12} + ...]$

	U^{11}	U^{22}	U^{33}	U^{23}	U^{13}	U^{12}
O(1)	11(1)	5(1)	8(1)	2(1)	5(1)	2(1)
O(2)	11(1)	6(1)	7(1)	3(1)	5(1)	2(1)
H(2)	23(1)	15(1)	25(1)	8(1)	10(1)	6(1)
O(3)	11(1)	5(1)	5(1)	0(1)	3(1)	2(1)
O(4)	11(1)	5(1)	7(1)	2(1)	5(1)	2(1)
N(1)	8(1)	5(1)	6(1)	1(1)	3(1)	2(1)
C(2)	7(1)	4(1)	5(1)	2(1)	3(1)	2(1)
C(3)	8(1)	4(1)	6(1)	1(1)	3(1)	1(1)
H(3)	31(1)	13(1)	17(1)	0(1)	10(1)	7(1)
C(4)	7(1)	5(1)	5(1)	1(1)	2(1)	2(1)
C(5)	9(1)	5(1)	5(1)	1(1)	3(1)	2(1)
H(5)	29(1)	20(1)	12(1)	5(1)	11(1)	8(1)
C(6)	9(1)	5(1)	6(1)	1(1)	3(1)	2(1)
H(6)	32(1)	13(1)	17(1)	-2(1)	10(1)	6(1)
C(7)	8(1)	6(1)	4(1)	1(1)	2(1)	3(1)
C(8)	11(1)	6(1)	10(1)	2(1)	4(1)	3(1)
H(8A)	24(1)	25(1)	32(1)	0(1)	14(1)	11(1)
H(8B)	42(1)	14(1)	28(1)	8(1)	18(1)	5(1)
H(8C)	25(1)	25(1)	20(1)	1(1)	-1(1)	10(1)

Table S6. Torsion angles [°] for MPANO.

O(1)-N(1)-C(2)-C(3)	179.95(7)
C(6)-N(1)-C(2)-C(3)	0.17(10)
O(1)-N(1)-C(2)-C(7)	1.31(10)
C(6)-N(1)-C(2)-C(7)	-178.47(6)
N(1)-C(2)-C(3)-C(4)	-0.40(11)
C(7)-C(2)-C(3)-C(4)	178.23(7)
C(8)-O(4)-C(4)-C(3)	2.26(13)
C(8)-O(4)-C(4)-C(5)	-178.28(7)
C(2)-C(3)-C(4)-O(4)	-179.85(8)
C(2)-C(3)-C(4)-C(5)	0.71(11)
O(4)-C(4)-C(5)-C(6)	179.71(8)
C(3)-C(4)-C(5)-C(6)	-0.80(11)
O(1)-N(1)-C(6)-C(5)	179.95(7)
C(2)-N(1)-C(6)-C(5)	-0.26(10)
C(4)-C(5)-C(6)-N(1)	0.58(11)
N(1)-C(2)-C(7)-O(3)	177.26(7)
C(3)-C(2)-C(7)-O(3)	-1.37(11)
N(1)-C(2)-C(7)-O(2)	-1.68(11)
C(3)-C(2)-C(7)-O(2)	179.69(7)

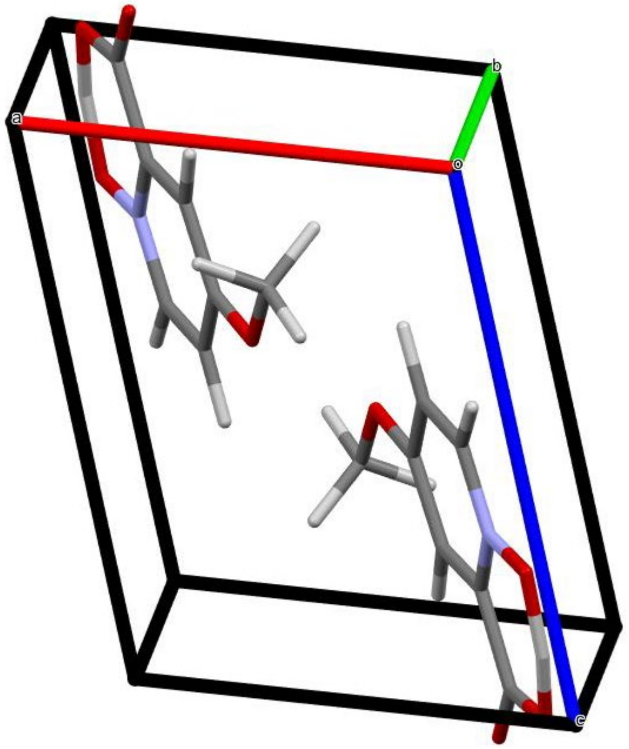

Figure S1. MPANO, unit cell content

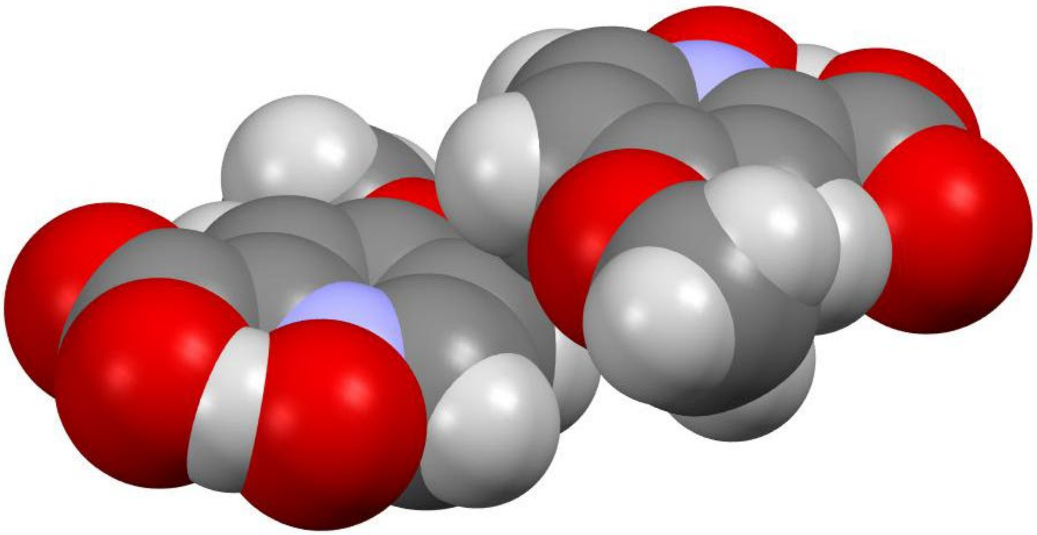

Figure S2. MPANO, space filling model of the unit cell content.

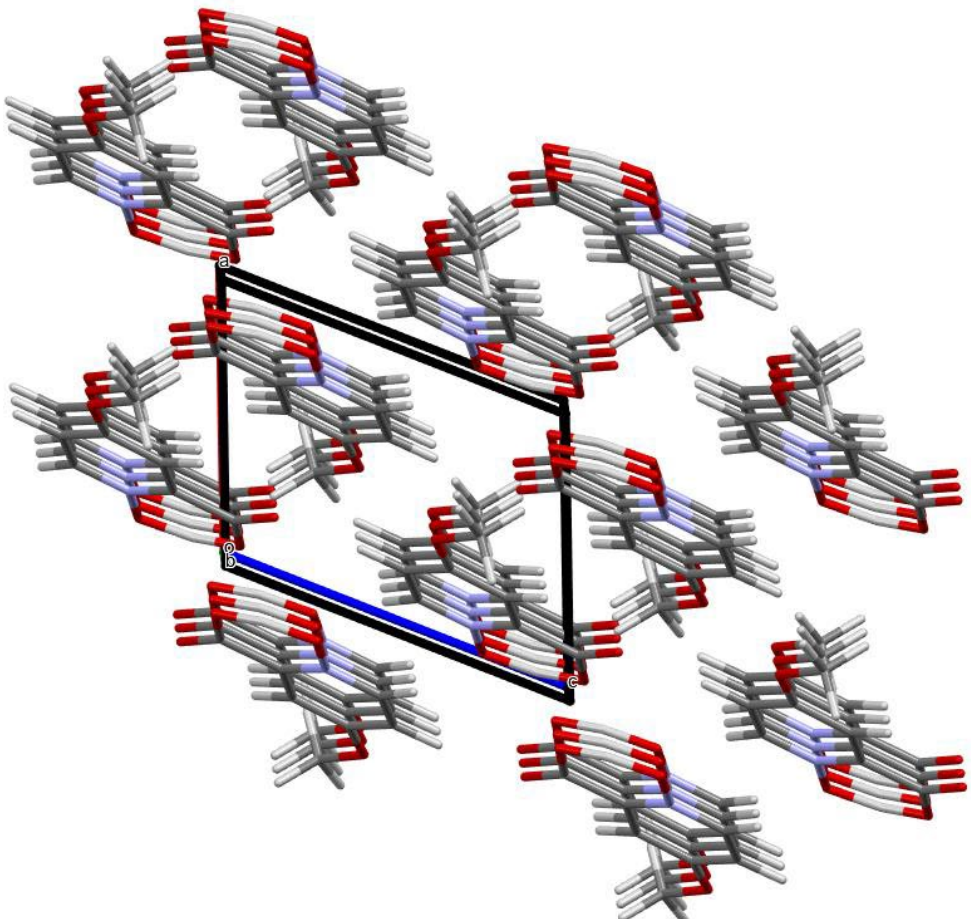

Figure S3. MPANO, molecular packing along the *b* axis

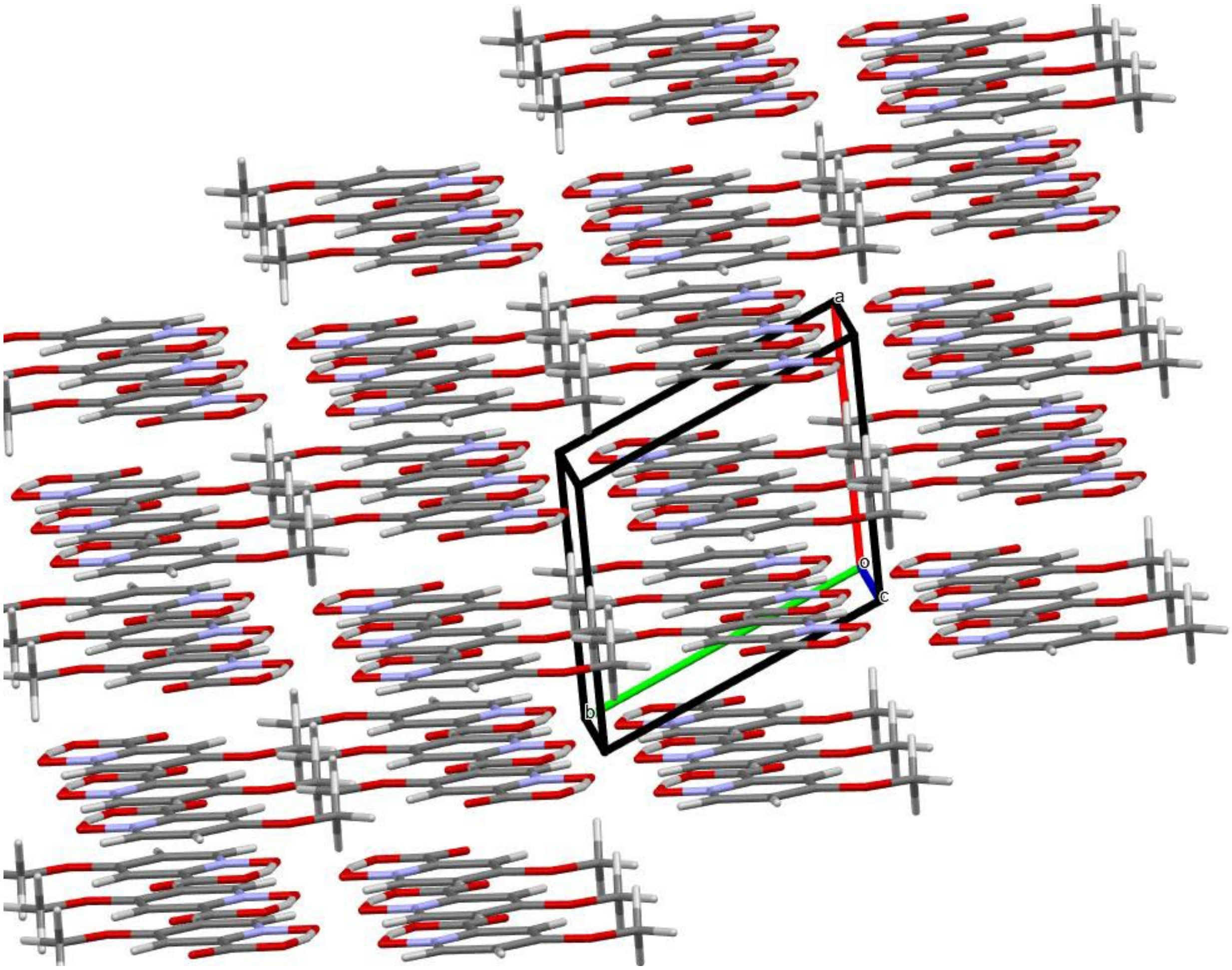

Figure S4. MPANO, molecular packing along the *a* axis.

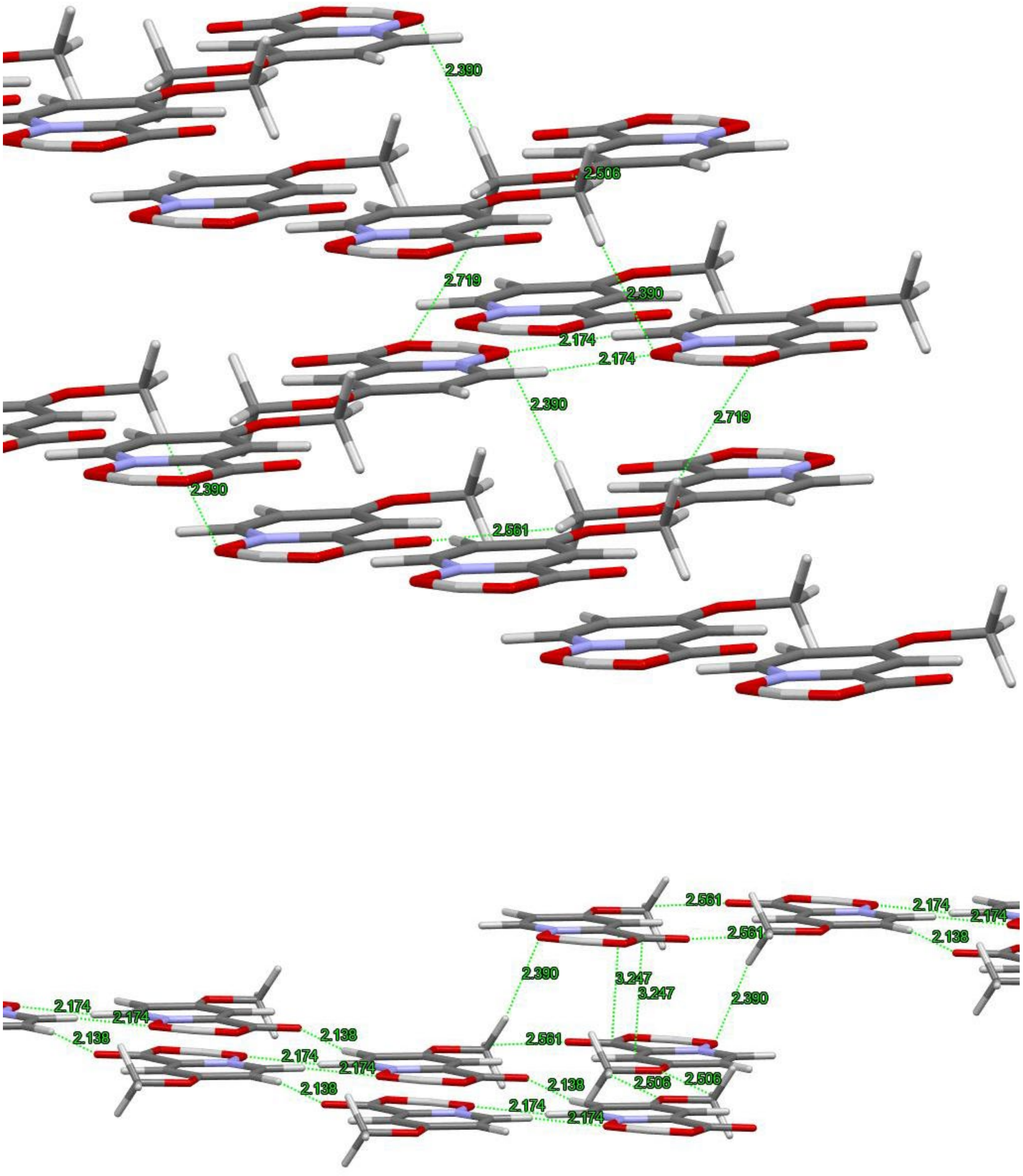

Figure S5. Significant packing distances for MPANO.

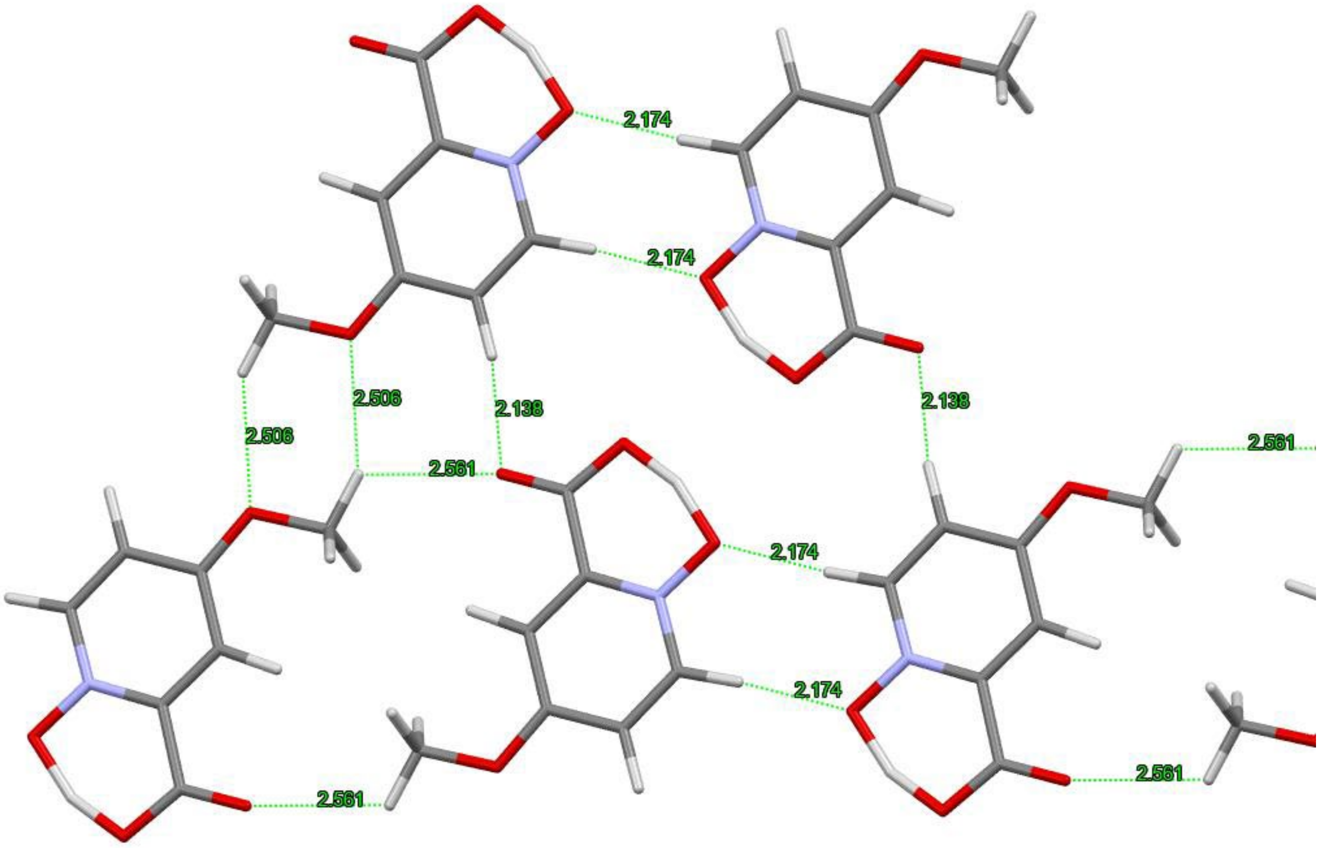

Figure S6. Significant intralayer distances for MPANO.

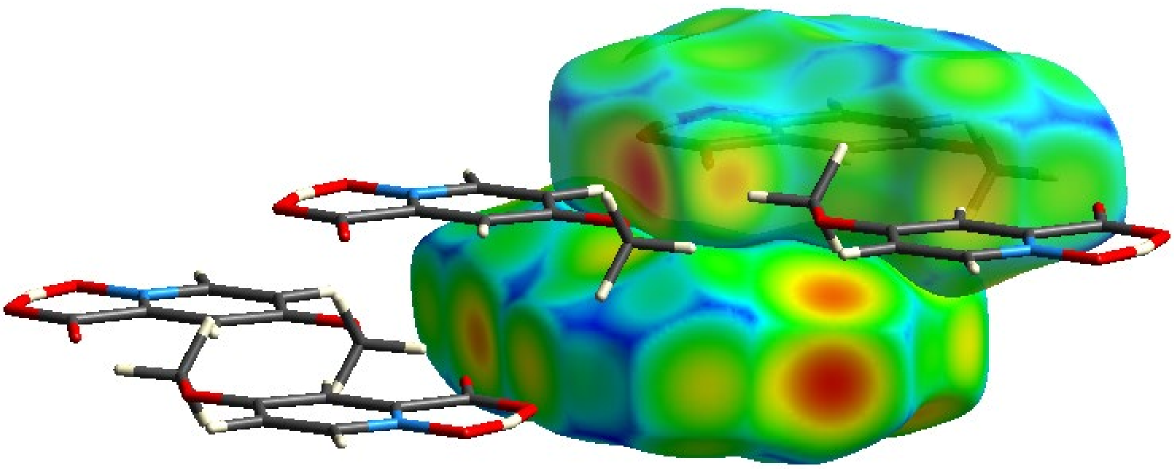

Figure S7. Hirshfeld surfaces of MPANO unit cell content mapped with d_i.

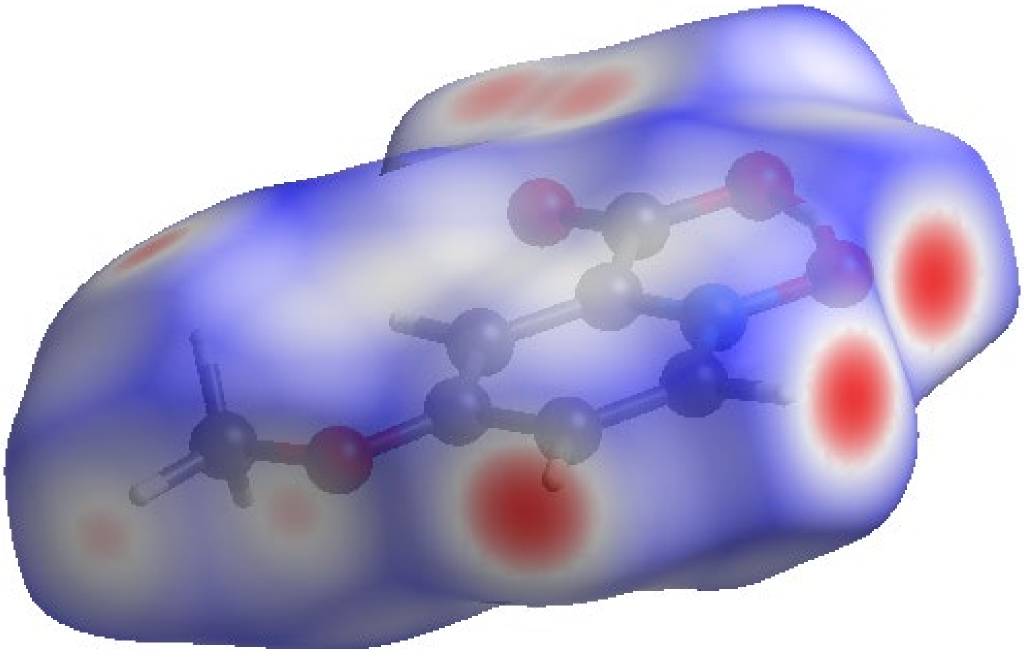

Figure S8. Hirshfeld Surfaces of MPANO mapped with d_{norm}.

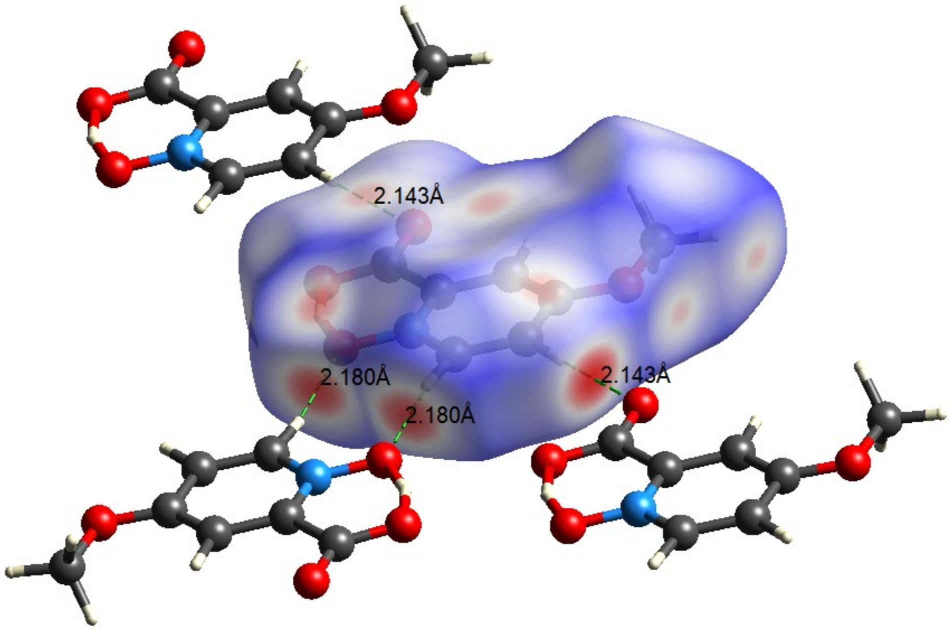

Figure S9. Hirshfeld Surfaces of MPANO mapped with d_{norm} , showing the hydrogen bond contacts in the 2D layers.

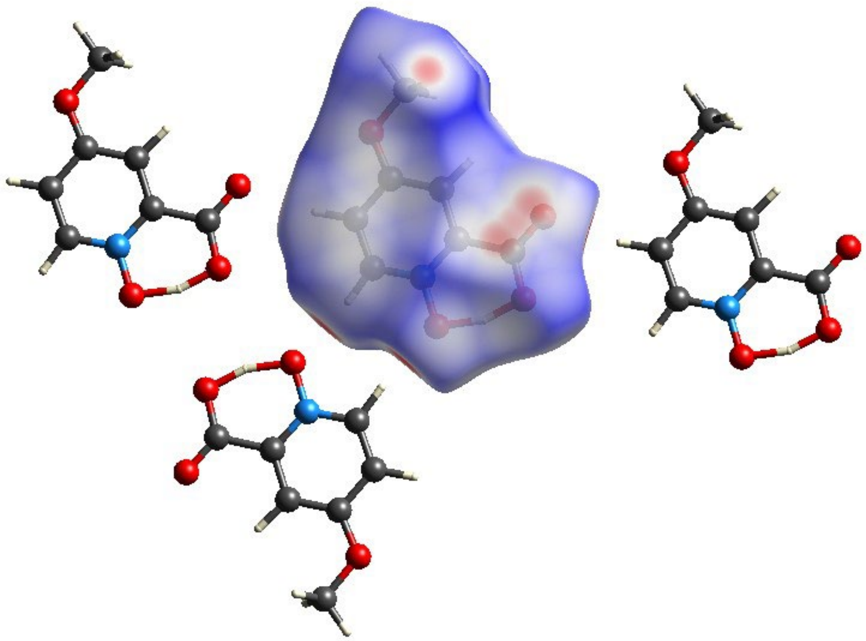

Figure S10. Same view as in Fig. S9, but perpendicular to the layers.

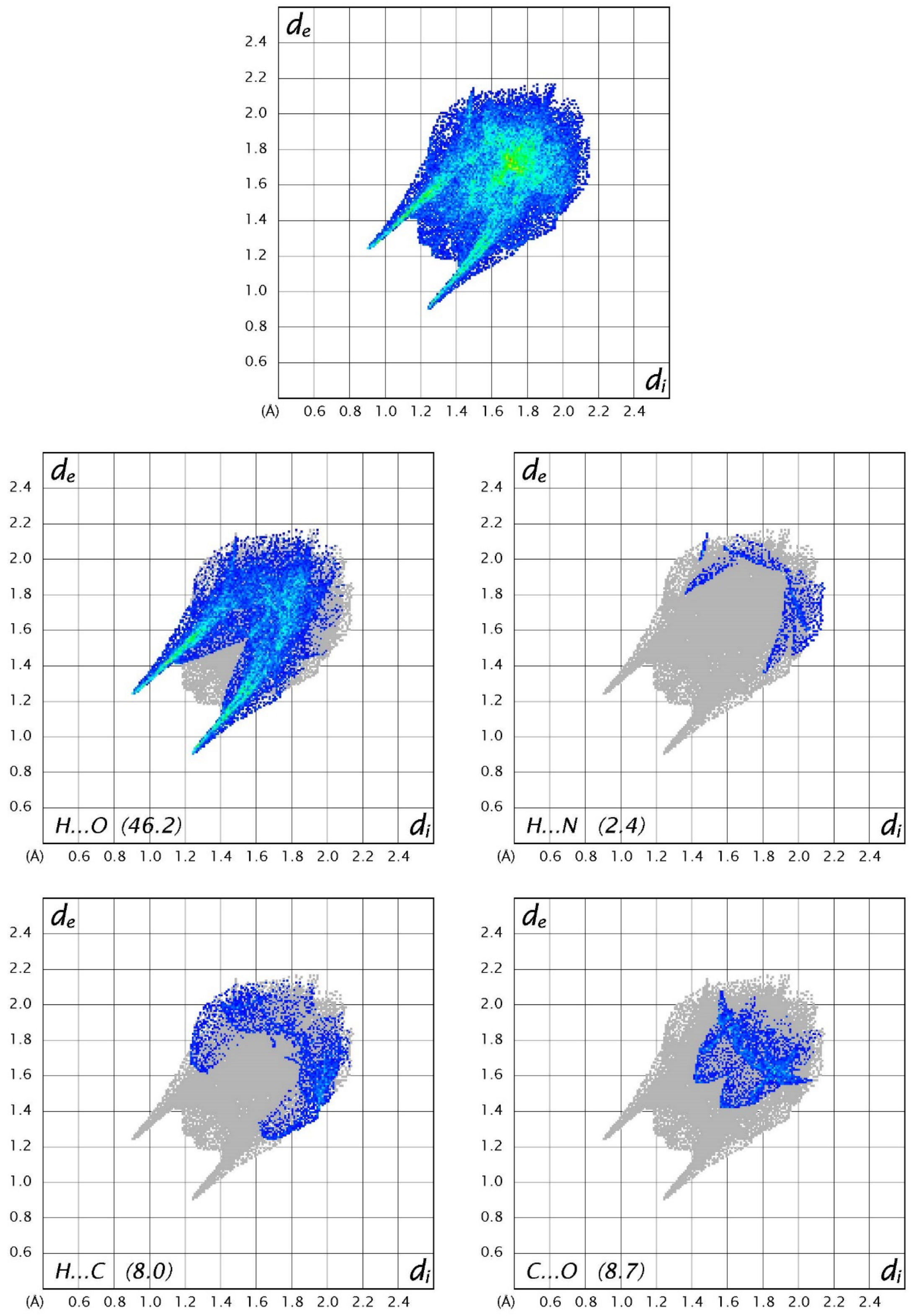

Figure S11. Fingerprint plot for MPANO (top line) and fingerprints resolved into H...O (middle line, left), H...N (middle line, right), H...C (bottom line, left) and C…O (bottom line, right) contacts (π-π). The full fingerprint appears beneath each decomposed plot as a gray shadow, the numbers in parentheses are the surface area included for each interaction (%).

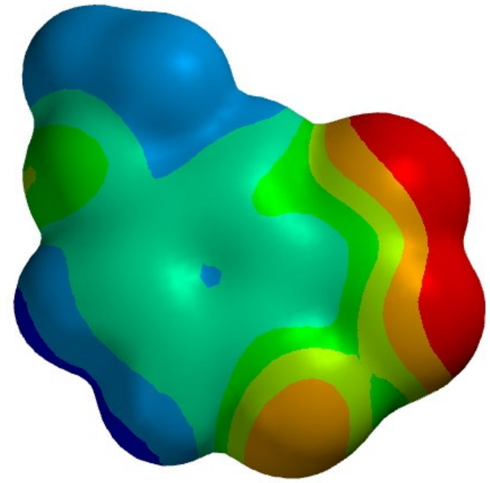

Figure S12. Plot of the electrostatic potential in MPANO

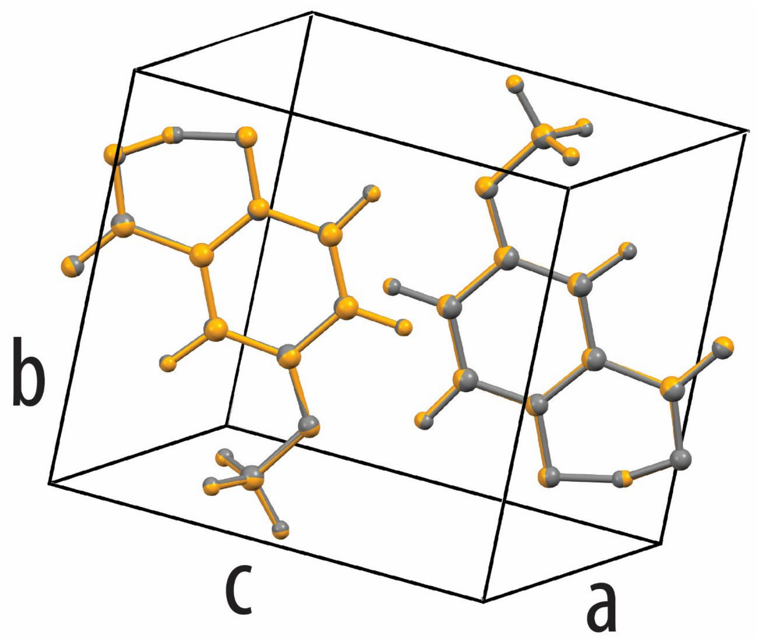

Figure S13. Overlaid unit cells of MPANO obtained from diffraction (grey) and from geometry optimization by periodic DFT (orange).

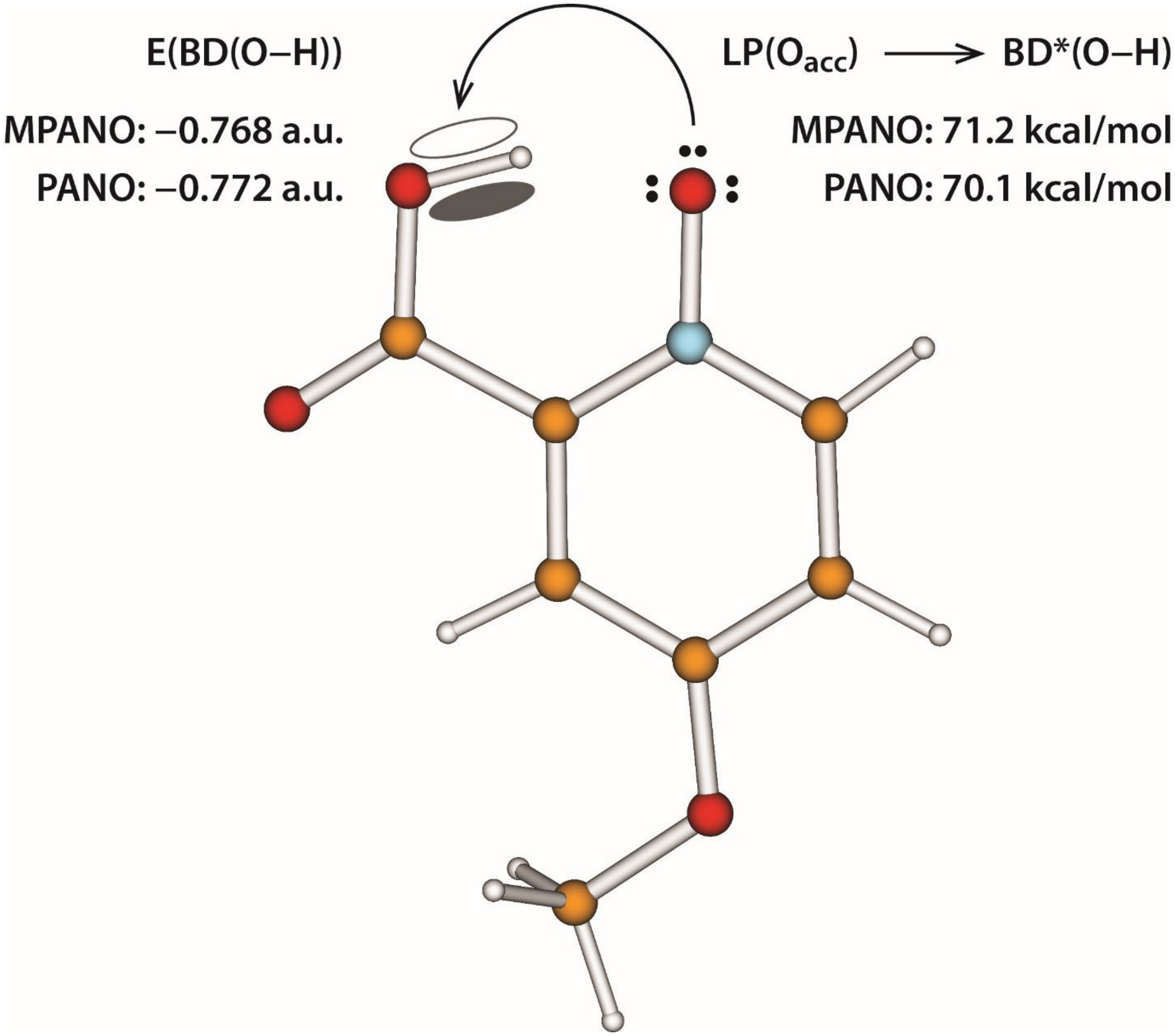

Figure S14. Structure of MPANO with schematic representation of orbitals and orbital interactions evaluated by the NBO method. Comparison of characteristic NBO quantities between MPANO and PANO is given. Note that in the part of structure common to MPANO and PANO (i.e. the entire molecule except for the methoxy / hydrogen substituent at ring position 4) the two molecules are in exactly the same geometry to ensure comparability, in that any difference in the electronic structure originates from the different nature of substituent at position 4.

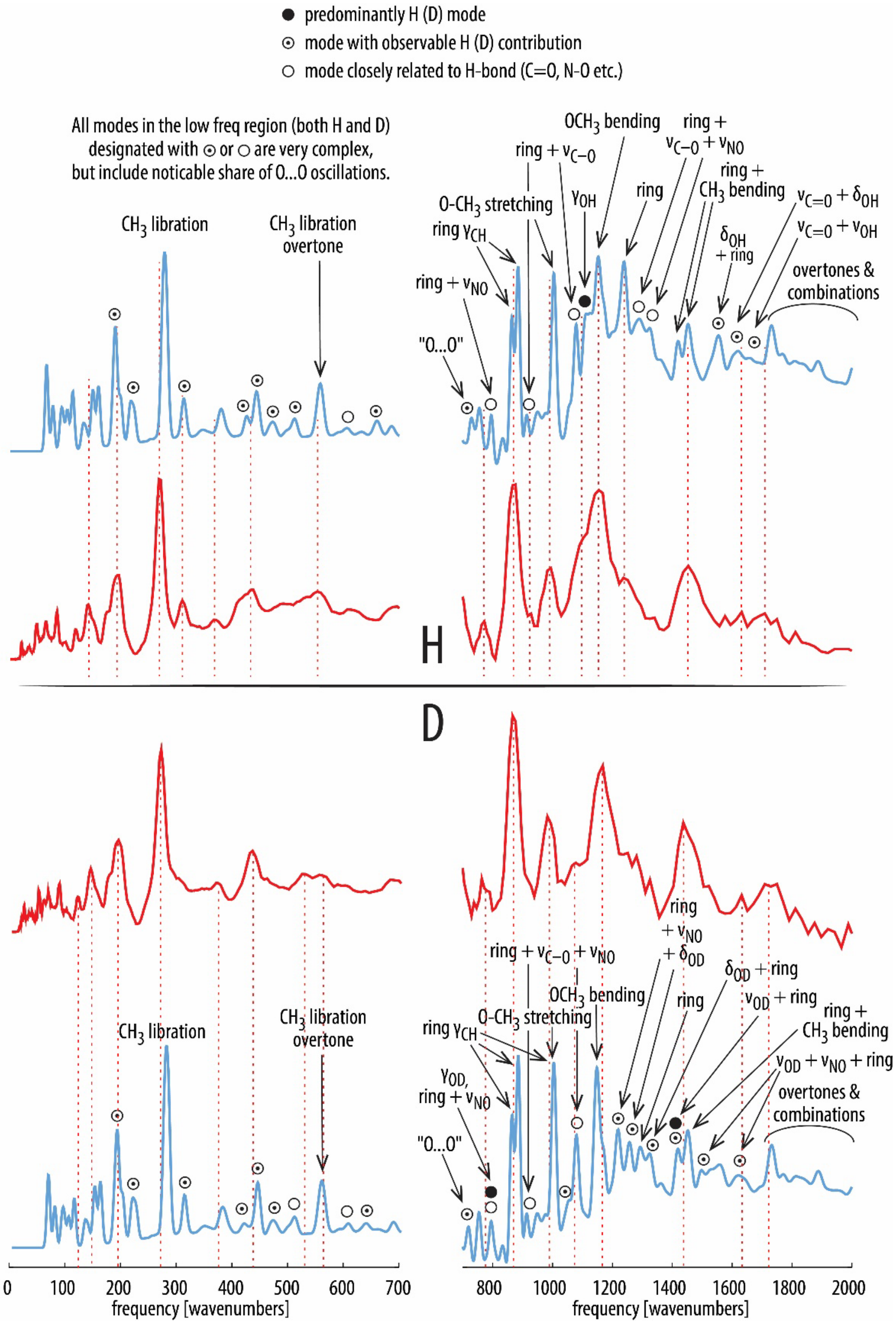

Figure S15. Assigment of INS spectrum of protic (top half) and H-bond deuterated (bottom half) MPANO based on visualization of computed normal modes using a fully periodic model. Calculated spectra are plotted in blue whereas the measured ones are shown in red. Vertical dashed red lines correspond to the peaks of selected bands in the experimental spectrum. Greek letters ν, δ and γ denote stretching, in-plane bending and out-of-plane bending modes, respectively. Note that the y-axis of the plots corresponds to INS intensity given in arbitrary units.

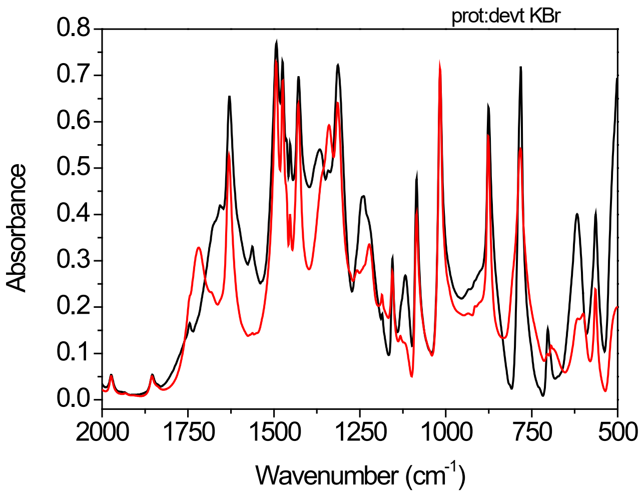

Figure S16. Comparison of the spectra upon the H/D exchange in the fingerprint region. The black spectrum corresponds to protic and red one to deuterated MPANO.

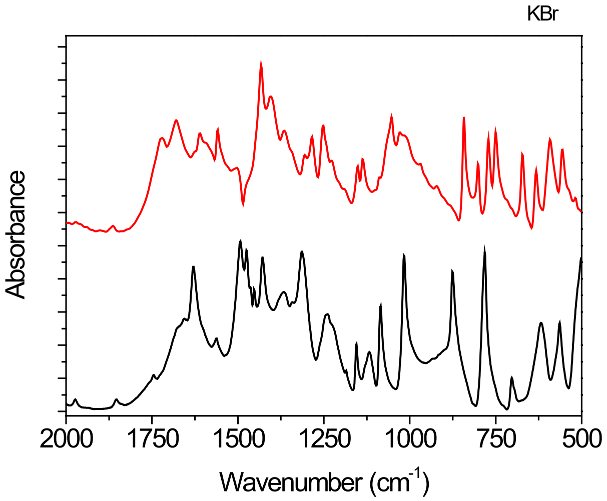

Figure S17. Comparison between PANO (red) and MPANO (black) infrared spectrum in the fingerprint region.

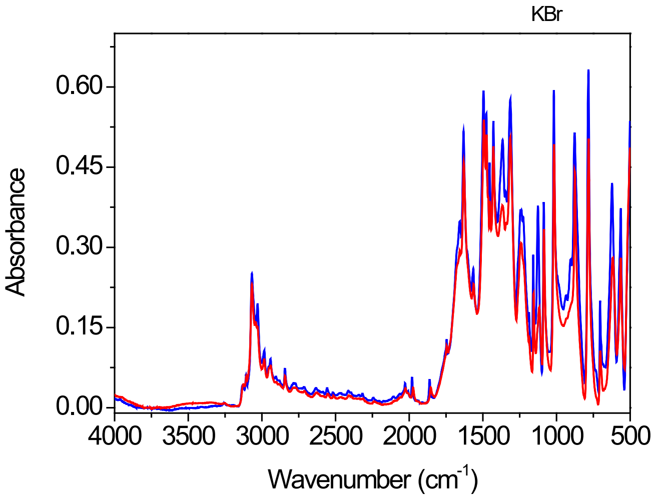

Figure S18. Infrared spectra of protic MPANO recorded at T=21 °C (red spectrum) and T=-150 °C (blue spectrum).

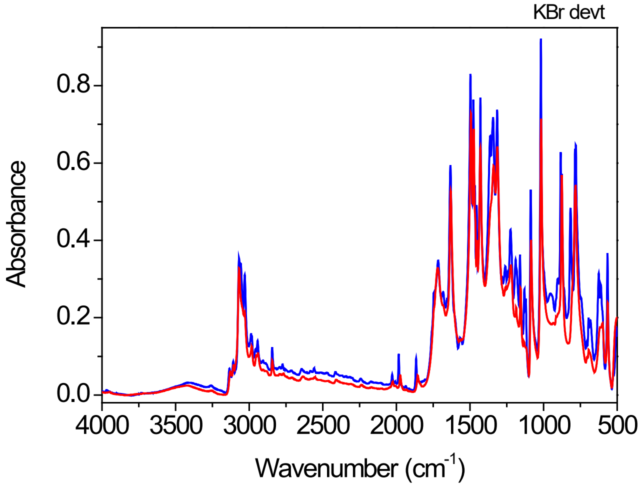

Figure S19. Infrared spectra of deuterated MPANO recorded at T=21 °C (red spectrum) and T=-150 °C (blue spectrum).
